# Supplementary material for: MetaQTL: a package of new computational methods for the meta-analysis of QTL mapping experiments
Source: BMC Bioinformatics. 2007 Feb 8;8:49. doi: 10.1186/1471-2105-8-49 (PMC1808479; doi:10.1186/1471-2105-8-49)
Supplement: Additional File 7 — MetaQTL Package : jar file and tutorial. This Zip archive contains both the MetaQTL JAR file and the files of the tutorial. [file 1471-2105-8-49-S7.zip › org.inra.metaqtl/doc/org/thalia/bio/IBioEntityTypes.html]

IBioEntityTypes


|  |  |  |
| --- | --- | --- |
| **Overview** \| **Package** \| **Class** \| **Use** \| **Tree** \| **Deprecated** \| **Index** \| **Help** | |  |
| **PREV CLASS**   **NEXT CLASS** | **FRAMES**    **NO FRAMES**     **All Classes** |  |
| SUMMARY: NESTED \| FIELD \| CONSTR \| METHOD | DETAIL: FIELD \| CONSTR \| METHOD |  |


---


## org.thalia.bio Interface IBioEntityTypes

---

``` public interface IBioEntityTypes ```

Class Description Here

**Author:**
:   Jean-Baptiste Veyrieras

---

| **Field Summary** | |
| --- | --- |
| `static int` | `ALLELE`             The integer code for Allele entity |
| `static int` | `COLLECTION` |
| `static int` | `GENOME`             The integer code for Genome entity |
| `static int` | `INDIVIDUAL`             The integer code for an individual entity |
| `static int` | `LGROUP`             The integer code for Linkage Group entity |
| `static int` | `LOCUS`             The integer code for Locus entity |
| `static int` | `MARKERLOCUS` |
| `static int` | `ONTOLOGY` |
| `static int` | `ONTOLOGY_TERM` |
| `static int` | `POPULATION`             The integer code for Population entity |
| `static int` | `QTLLOCUS` |

**Field Detail**

### INDIVIDUAL

```
static final int INDIVIDUAL
```

:   The integer code for an individual entity

    **See Also:**: Constant Field Values

---


### POPULATION

```
static final int POPULATION
```

:   The integer code for Population entity

    **See Also:**: Constant Field Values

---


### GENOME

```
static final int GENOME
```

:   The integer code for Genome entity

    **See Also:**: Constant Field Values

---


### LGROUP

```
static final int LGROUP
```

:   The integer code for Linkage Group entity

    **See Also:**: Constant Field Values

---


### LOCUS

```
static final int LOCUS
```

:   The integer code for Locus entity

    **See Also:**: Constant Field Values

---


### MARKERLOCUS

```
static final int MARKERLOCUS
```

**See Also:**: Constant Field Values

---


### QTLLOCUS

```
static final int QTLLOCUS
```

**See Also:**: Constant Field Values

---


### ALLELE

```
static final int ALLELE
```

:   The integer code for Allele entity

    **See Also:**: Constant Field Values

---


### COLLECTION

```
static final int COLLECTION
```

**See Also:**: Constant Field Values

---


### ONTOLOGY\_TERM

```
static final int ONTOLOGY_TERM
```

**See Also:**: Constant Field Values

---


### ONTOLOGY

```
static final int ONTOLOGY
```

**See Also:**: Constant Field Values


---


|  |  |  |
| --- | --- | --- |
| **Overview** \| **Package** \| **Class** \| **Use** \| **Tree** \| **Deprecated** \| **Index** \| **Help** | |  |
| **PREV CLASS**   **NEXT CLASS** | **FRAMES**    **NO FRAMES**     **All Classes** |  |
| SUMMARY: NESTED \| FIELD \| CONSTR \| METHOD | DETAIL: FIELD \| CONSTR \| METHOD |  |


---
